# The cholera paradox: Removing the pump handle

**DOI:** 10.4102/jphia.v15i1.751

**Published:** 2024-09-26

**Authors:** Emmanuel Agogo

**Affiliations:** 1 Global Health and Infectious Diseases Control Institute, Nasarawa State University Keffi, Keffi, Nigeria

Cholera is a marker of inequity, poverty, and a lack of social development. Unfortunately, the world has been unable to curtail the scourge of cholera for many centuries. The seventh pandemic of cholera has been ongoing since 1961. Post-coronavirus disease 2019 (COVID-19), there has been a spike in cholera cases, with 473 000 cholera cases reported to the World Health Organization (WHO) in 2022, which is double the number from 2021. A further increase to 700 000 cases was estimated for 2023.^1^ In 2024, almost 30 countries have reported cholera outbreaks with the most severe impacts in Comoros, Democratic Republic of Congo, Ethiopia, Mozambique, Somalia, Zambia, and Zimbabwe. During the first 6 months of 2024, a cumulative total of 249 793 cholera cases and 2137 deaths were reported across five WHO regions, with the Eastern Mediterranean Region recording the highest numbers, followed by the African Region (worst outcome for the cases on the African continent), the region of the Americas, the South-East Asia region, and the European region (see Table 1).^2^

**TABLE 1:** Global cholera cases summarised by continental burden.

Continent	Cases	Deaths	CFR (%)
**Africa**	111 938	1822	1.63
Uganda	-	-	5.60
Sudan	-	-	2.70
Zambia	-	-	3.20
Middle East	122 500	148	0.12
Europe	210	2	1.00
Americas	2672	13	0.49
Asia	2250	6	0.27

*Source*: Adapted from WHO. Multi-country outbreak of cholera; External Situation Report no. 16, published 18 July 2024. Geneva: World Health Organization; 2005

CFR, case fatality ratio.

The Global Health Security (GHS) conventions that now guide global pandemics have deep historical roots in the International Health Regulations (IHR) 2005, which in turn were underpinned by the series of 14 International Sanitary Conferences held since 1851 aimed at standardising international quarantine regulations against the spread of cholera, plague, and yellow fever.^3^ Over time, the global implementation of Joint External Evaluations (JEEs) and other indices such as the Health Security Index have helped assess country preparedness to prevent, detect, and respond to public health events such as cholera. However, these assessments have revealed a persistent mismatch between these evaluations and actual country preparedness, as evidenced by the global COVID-19 response. This mismatch calls for re-evaluating how we measure and implement GHS interventions.^4^

New iterations of the JEE tool now include the availability of basic infrastructure, including access to water and sanitation facilities in health institutions, highlighting the intersection of GHS and hygiene within the health system. These interventions stem from recognising improved infection prevention and control measures in disease prevention and the spread of disease within healthcare infrastructure. Other concepts that have gained importance in the new iteration of the country JEE assessments include provisions addressing healthcare-associated infections (HCAIs), community-focused risk communication, cross-border surveillance, early warning systems for outbreaks, and adopting a One Health approach.

The One Health concept connects human health, animal health, and environmental health, offering a comprehensive strategy to tackle infectious disease outbreaks such as cholera, antimicrobial resistance (AMR), and other global health threats. However, the environmental sector and health systems interventions aimed at addressing environmental preparedness and surveillance have consistently remained the weakest. This is the case despite the emphasis on water, sanitation, and hygiene (WASH) in the sustainable development goals (SDGs) at the community level. Significant gaps remain in global WASH standards, which impact health outcomes. Although the SDGs recognise the importance of interventions needed to fight diseases associated with poverty, it is essential to make a bold shift towards comprehensive solutions.

To address the significant GHS challenges, it is important to consider unconventional and more broad-based approaches that go beyond the boundaries of the healthcare system, especially in combating cholera. This new approach to global health changes the focus to prevention, fairness, and overall well-being as the way to address the world’s most urgent health challenges.

Lessons from COVID-19 are invaluable. Key public health and social measures (PHSMs) implemented during the pandemic have shown the importance of preventive strategies. Despite the challenges of implementation, these measures – such as ensuring access to potable water, implementing environmental controls to limit the spread of infection, and enforcing effective isolation and contact tracing – played a crucial role in saving lives. Additionally, the use of preventive personal equipment, prompt case identification, and supportive treatment are also essential components of a robust response to health emergencies. These foundational practices, proven effective during COVID-19, are equally critical for controlling other infectious diseases like cholera.

In addition, there is a lack of a coordinated whole-of-government and whole-of-society approach needed to tackle the persistent cholera crisis in Africa. This deficiency hampers efforts to mobilise resources and address the inadequate public infrastructure that exacerbates the suffering and mortality among the world’s poorest and most neglected communities.

Bold community efforts can make a difference. Universal access to clean water can eradicate waterborne and faecal–oral transmitted diseases, marking a significant leap in global health. Good governance and the rule of law are essential for fostering fairness, and justice, and eradicating corruption, ultimately improving health outcomes. Ensuring access to adequate shelter and sanitation promotes healthy living environments and reduces the transmission of diseases. Addressing food safety concerns and ensuring adequate nutrition can halt the spread of foodborne diseases and combat malnutrition. Meaningful employment and universal income guarantee economic stability and reduce health disparities.

However, this paradigm shift poses challenges to certain vested interests and power structures. The medical–industrial complex, heavily invested in pharmaceuticals and medications, may face challenges as the focus shifts towards prevention rather than treatment. The humanitarian complex, reliant on traditional aid models, may need to adapt to this new approach. Corrupt governments and leaders, whose power is rooted in inequity and corruption, may feel threatened by the transparency and equity this shift demands. Aid agencies, traditionally focused on medical assistance, may need to pivot towards broader development goals.

Regional perspectives, such as the aspirations of the African Union’s Agenda 2063, provide context and direction for these transformative changes. By aligning with regional goals and narratives, a holistic approach to global health can be both globally relevant and locally impactful.

In conclusion, addressing GHS challenges requires a radical rethink of our current strategies. By prioritising prevention, fairness, and overall well-being, we can create sustainable solutions that not only combat diseases such as cholera but also promote overall GHS.
